# Cardiovascular Risk Factors Promote Brain Hypoperfusion Leading to Cognitive Decline and Dementia

**DOI:** 10.1155/2012/367516

**Published:** 2012-12-03

**Authors:** Jack C. de la Torre

**Affiliations:** Department of Psychology, University of TX at Austin, Austin, TX 78712, USA

## Abstract

Heart disease is the major leading cause of death and disability in the world. Mainly affecting the elderly population, heart disease and its main outcome, cardiovascular disease, have become an important risk factor in the development of cognitive decline and Alzheimer's disease (AD). This paper examines the evidence linking chronic brain hypoperfusion induced by a variety of cardiovascular deficits in the development of cognitive impairment preceding AD. The evidence indicates a strong association between AD and cardiovascular risk factors, including ApoE_4_, atrial fibrillation, thrombotic events, hypertension, hypotension, heart failure, high serum markers of inflammation, coronary artery disease, low cardiac index, and valvular pathology. In elderly people whose cerebral perfusion is already diminished by their advanced age, additional reduction of cerebral blood flow stemming from abnormalities in the heart-brain vascular loop ostensibly increases the probability of developing AD. Evidence also suggests that a neuronal energy crisis brought on by relentless brain hypoperfusion may be responsible for protein synthesis abnormalities that later result in the classic neurodegenerative lesions involving the formation of amyloid-beta plaques and neurofibrillary tangles. Insight into how cardiovascular risk factors can induce progressive cognitive impairment offers an enhanced understanding of the multifactorial pathophysiology characterizing AD and ways at preventing or managing the cardiovascular precursors of this dementia.

## 1. Introduction

It has been known since the Ebers papyrus [1] in 1552 BC, and probably even before then, that the brain and heart are intimately connected. The ancient Greeks and Aristotle in particular believed that the function of the brain was to “cool” the blood while the heart was the source of memory. This belief was consolidated by religious and scientific dogma for centuries. It took the most significant achievements in medicine in the 16th and 17th centuries by the Belgian anatomist Andreas Vesalius and the English physician William Harvey to challenge that prevailing dogma and describe a more accurate account of the cerebral circulation as well as the heart's continuous pumping action inside a very precise circuit.

Fast-forwarding to the 20th century, several researchers in the late 1970s became aware of an intriguing link between a sick heart and the start of cognitive deterioration that often led to vascular dementia (VaD) [2]. This link came to be known as “cardiogenic dementia”, and although it remained largely ignored for many years as a casual observation, it eventually opened the door slightly to a fascinating field where cognitve impairment and dementia could be triggered by a bad heart [2].

Research in the early 1990s additionally began to suggest that cardiovascular disease could also signal the start of Alzheimer's disease (AD), an assumption that, as will be seen below, has gained evidence-based support from more detailed studies published in the last decade. The suggested link between cardiovascular deficits as a risk to AD was important not only because it has now been generally accepted to be true but because it implied that other extracardiac vascular risk factors might also play a crucial role in the initiation of this dementia by possibly sharing common pathological pathways or markers common in AD. Moreover, the therapeutic implications of controlling such vascular risk factors to AD have become one of the most important inroads in the search to lower the rising prevalence of AD and VaD [3, 4]. 

Cardiovascular disease comes in many forms, and its outcome is dependent on the patient's age, prior history, life style, primary prevention, genetics and pathological factors affecting structural, and hemodynamic function. Cardiovascular disease affects the heart vessels which affect the structural tissue of the heart and can be classified as coronary (the most common), valvular, cardiomyopathic, arrhythmic, congenital, and heart failure [5]. Due to space constraints, only some forms of cardiovascular disease will be reviewed here although it is anticipated that most forms of heart disease which lower cerebral blood flow through an impairment in cardiac structure or physiology will also be found to increase AD risk. 

## 2. Cardiovascular Disease, Cognition, and Cerebral Autoregulation

The original association between cardiac pathology and cognitive dysfunction described above as cardiogenic dementia was based on the high incidence of cardiac dysrhythmias seen in patients with dementia solely due to vascular causes [6]. Thus, chronic heart block and dysrhythmias that were shown to lower cardiac output and lead to persistent cognitive dysfunction and dementia were rightly believed to stem from a diseased heart [6]. When arrhythmias were suspected to cause cognitive dysfunction, it was found that cognitive dysfunction could be attenuated or reversed with cardiac pacing [6, 7]. Restoration of cognitive ability by pacing was attributed to bringing the impaired cerebral perfusion back to normal by maintaining an adequate heart rate [6].

At the present time, brain hypoperfusion that develops from low cardiac output or hypotension has been shown to cause cognitive deficits in attention and memory [8], and more recent studies propose that these deficits develop from brain hypoperfusion and can lead to AD [9–11]. 

When hypotension is involved, its link to cognitive decline has been generally ignored in clinical practice. One reason for this attitude is the current dogma that low systemic blood pressure does not cause brain dysfunction because compensatory cerebral autoregulation prevents brain hypoperfusion from being activated [12]. However, studies have confirmed, particularly in the elderly, that cerebral autoregulation does not necessarily protect the brain from chronic low blood pressure and low cardiac output, an outcome that can result in cerebral blood flow insufficiency and its accompanying consequences [9, 13–15]. 

Evidence to support the concept linking cognitive function to cerebral perfusion also comes from studies showing that cerebral blood flow rises in healthy subjects following moderate exercise but cerebral perfusion may not increase if cardiac output is limited by cardiac pathology affecting, for example, heart rate from a dysrhythmia [16]. 

These findings indicate that cerebral autoregulation does not reverse brain hypoperfusion when cardiac output is compromised by specific cardiac pathology. Normally, cerebral autoregulation maintains a constant blood flow to the brain between 50–150 mm Hg mean arterial pressure. The main function of cerebral autoregulation is to maintain cerebral perfusion pressure by signaling brain arterioles to dilate or constrict during changes in arterial blood pressure [17]. Cerebral autoregulation may become impaired with aging [18] but it is not clear why or how. The long-term evolution for potential brain damage during aging can involve decades. It may develop from atherosclerosis, arterial stiffness, or cardiovascular disease which can impair cerebral autoregulation homeostasis and result in brain hypoperfusion (Figure 2). Brain hypoperfusion would preferentially affect the older brain which is already primed to neurometabolic dysfunction by decreased delivery of energy substrates to brain cells [19–21]. 

Studies have reported that normal aging reduces CBF about 20% at age 60 as compared to age 20 [22–26], so any additional burden which further lowers cerebral perfusion in addition to that seen during aging could damage or kill vulnerable neurons [19, 27]. Glucose is the primary molecule used to create energy fuel for mammalian brain cells and the brain depends on a continuous and optimal flow of blood to maintain normal brain cell activity and structural integrity [28].

If impaired cerebral autoregulation is indeed one of the culprits that contributes to cognitive failure in the elderly following chronically evolving vascular insults, a number of cerebral vasoactive molecules may be coconspirators in the development of AD, among them vasodilators such as endothelial nitric oxide, adenosine, prostacyclin, and epoxygenases that yield epoxyeicosatrienoic acids and vasoconstrictors such as thromboxane A_2_, endothelin-1, and 20-hydroxyeicosatetraenoic acids (20-HETE). Curiously, pharmacologic inhibition of 20-HETE appears to impair cerebral autoregulation *in vivo* [29]. 

Another cyclic burden on the heart that generates reduced blood flow to the brain is the finding that cerebral hypoperfusion can impair cerebrovascular reactivity [30, 31], possibly as a result of reduced nitric oxide release from damaged endothelial cells [32]. 

Other vascular risk factors, such as chronic hypertension can shift the limits of autoregulation toward higher blood pressure levels [33]. This adaptative mechanism protects the brain against hypertension to some degree but can also render it more vulnerable to cerebral hypoperfusion in elderly individuals who are aggressively treated with antihypertensive medication that induces hypotension. For this reason, it has been argued that careful consideration should be exercised when elderly persons with mild or moderate hypertension are given anti-hypertensive therapy, since such a treatment may lead to unregulated hypotension and risk of dementia [34, 35]. 

Not surprisingly, when dynamic cerebral autoregulation is impaired from unilateral carotid artery stenosis, carotid endarterectomy, or stenting can reverse the effects of the brain dysautoregulation [36]. Recent evidence indicates that patients undergoing carotid artery stent placement for atherosclerosis showed either neurocognitive improvement at the end of 12 months or no worsening in status from baseline, suggesting that this procedure is safe and possibly effective [37]. Cerebral autoregulatory dysfunction can also occur with vertebral artery disease although little can be done surgically to correct impaired blood flow from these lesioned vessels [38].

**Figure 1:**
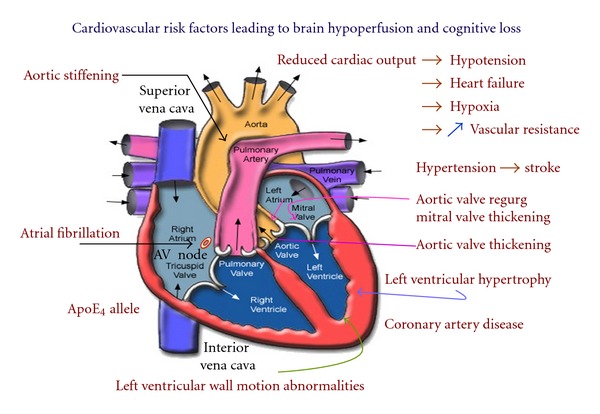
Cardiovascular risk factors reported to promote progressive cognitive decline leading to dementia in the elderly population. Reduced cardiac output (<3.4 ± 0.5 L/min) in the absence of clinically identified stroke can promote hypotension, heart failure, hypoxia, and increased (↑) vascular resistance associated with cognitive decline [39, 42, 63, 64] and ostensibly, Alzheimer's disease [173]. Aortic and mitral valve regurgitation (regurg) and/or valvular thickening can impair normal cardiac output. Aortic stiffening is associated with aging, hypertension, and atherosclerosis and is believed to result in brain microvascular damage, leading to cerebral hypoperfusion and cognitive decline [35, 118]. Left ventricular wall motion abnormalities mainly result from myocardial ischemia. Atrial fibrillation is a risk factor for cardioembolic events, especially stroke and for Alzheimer's disease [71]; it is the most common arrhythmia in the elderly population. Epidemiologic and clinical evidence indicates that coronary artery disease, the leading cause of mortality in the United States, is a potential risk factor for Alzheimer's disease [78, 109]. Left ventricular hypertrophy may be asymptomatic, mild, moderate, or severe and is a reported risk factor to cognitive decline in middle age but loses its predictive value in advanced age [174]. Presence of the ApoE_4_ allele, a genetic risk factor to Alzheimer's dementia, increases the risk of coronary heart disease by about 40% [114]. Presence of two or more cardiovascular risk factors may significantly accelerate the onset of cognitive deficits [161].

Despite the obvious importance that connects some types of heart disease to cognitive impairment (Figure 1), it has been largely ignored by many researchers in the field and by most cardiologists as evidenced by the absence of presentations discussing this topic in Alzheimer or heart-related conferences. We are aware of scant few papers that have questioned whether cognitive decline generated by low cardiac output in the mildly symptomatic elderly can lead to AD or VaD [39, 40]. Moreover, very few studies have examined the role of cardiac disease as a source of cerebral hypoperfusion [41–44] despite evidence that the presumed mechanism leading to cerebral hypoperfusion secondary to reduced cardiac output is left ventricular dysfunction associated with a reduced stroke volume [45]. 

The ultimate fallout from low cardiac output on the brain is that hemodynamic pump dysfunction in the older person can significantly lower blood flow to brain cells via cerebral hypoperfusion, thereby diminishing energy substrate supply needed for normal brain cell metabolism [46–50].

## 3. Cardiovascular Disease as a Vascular Risk Factor to Alzheimer's Disease

**Figure 2:**
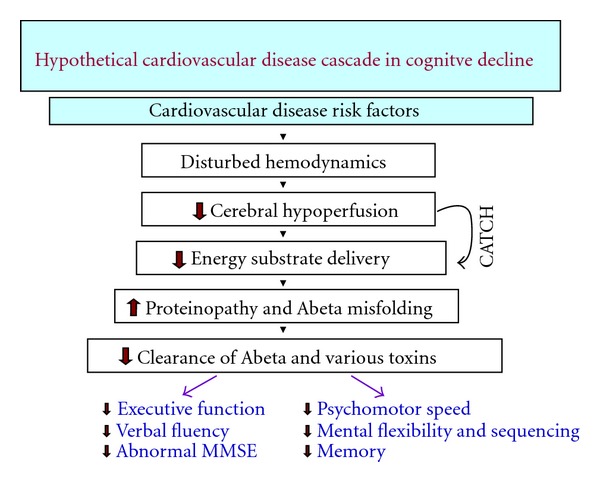
Hypothetical model based on the collective evidence available showing how cardiovascular risk factors give rise to disturbed hemodynamic flow patterns inducing cerebral hypoperfusion. Chronic insufficiency of blood flow to the brain may reach a critically attained threshold of cerebral hypoperfusion (CATCH) [19] responsible for lowered energy substrate delivery and creation of a neurono-glial energy crisis, initially in brain regions where memory and learning are localized. Further downstream, an increase in protein pathology (↑ proteinopathy), featuring protein misfolding of Abeta peptide, ensues followed by impaired clearance of waste products including Abeta [175]. Reduced Abeta clearance from the brain is possibly due, as we predicted, to an impaired microcirculation causing an ineffective efflux of waste products [121]. Deficits (↓) of nonmemory executive function, verbal and mental abilities, and psychomotor speed are ostensibly the first subclinical changes in cognitive dysfunction prior to more advanced cognitive impairment.

Epidemiological findings indicate that a broad spectrum of cardiovascular risk factors, including heart failure, thrombotic events, hypertension, hypotension, homocysteine, hypercholesterolemia, C-reactive protein, coronary artery disease, valvular disease, heart failure, apoE_4_, and atrial fibrillation are more common in the elderly. These conditions are reported to contribute to cognitive dysfunction and decline affecting performance in executive functions, attention, learning, psychomotor speed, verbal fluency, mental alertness, and memory [51–60] (Figure 2). Consequently, the neuropathologic link between the cardiac abnormalities listed above and their satellite off-shoots, such as amyloid angiopathy and presence of the ApoE_4_ genotype [61], is seem to contribute to a vascular complex that appears to target AD via specific cardiopathic pathways. 

## 4. Low Ejection Fraction or Low Cardiac Output

Ejection fraction a measure of stroke volume based on the dimensions of the left ventricle which is the main pumping chamber and refers to the percentage of blood that is pumped out of a filled left ventricle with each heartbeat contraction. Cardiac output is a measure of stroke volume based on forward flow velocities reflecting the amount of blood exiting the heart, as measured by liters per minute.

There is mounting epidemiologic evidence that AD is associated with an increased risk of symptomatic left ventricular dysfunction (LVD) [62]. LVD produces many changes in the structure and function of the heart through a variety of mechanisms. It can lead to reduced ejection fraction, heart failure, heart attack, and other cardiovascular complications. 

Low ejection fraction or low cardiac output in elderly patients with heart failure is reported to be associated with impairments of specific aspects of attention, specifically continuous vigilance and discriminability [63]. Low cardiac output has been found to be associated with impairment of executive function, including sequencing and planning [64] (Figure 2).

Cardiac resynchronization therapy is a relatively recent intervention that has been shown to increase cardiac function in symptomatic heart failure resulting from systolic dysfunction. It does this essentially by improving cardiac hemodynamics, ventricular contractility, and stroke volume while reducing myocardial energy consumption [65]. This technique has shown usefluness in improving executive and visuospatial functioning and neurocognitive measures of attention in patients with low left ventricular ejection fraction as compared to similar patients who did not undergo cardiac resynchronization therapy [66, 67]. 

Age may be a determinant of potential cognitive dysfunction. When ejection fraction dropped below 30%, patients older than 63 years showed a significant decline in memory performance, specifically, verbal delayed recall and recognition as compared to those under 63 years who were able to maintain stable memory function or similar ejection fraction levels [68]. Although subtle cognitive changes may progress to global cognitive decline and dementia, this conclusion requires further work to prove or disprove. Nevertheless, it provides a preventive guide when clinical testing detects elderly patients with low ejection fraction or cardiac output. These findings show that advancing age, cardiovascular pathology, and cognitive function are closely linked, and that novel interventions to correct impending cardiac hemodynamic homeotassis could make a difference in preventing or significantly slowing a potential pathway to dementia.

## 5. Atrial Fibrillation

Atrial fibrillation is a heart rhythm disorder (arrhythmia) usually involving a rapid heart rate. In the normal heart, the rate of ventricular contraction is the same as the rate of atrial contraction. In atrial fibrillation, however, the rate of ventricular contraction is less than the rate of atrial contraction. This condition can lead to a decrease in cardiac output diminishing the amount of blood pumped into the body by the ventricles because the atria are unable to fill the ventricles adequately due to their rapid rate of contraction and their absence of normal contractions. The risk of atrial fibrillation increases with age and is more common in males [69]. 

Not surprisingly, studies have shown an association between atrial fibrillation and diminished cognitive function leading to AD in the absence of stroke, high blood pressure, and diabetes [70]. The risk of AD after atrial fibrillation has been reported stronger than for vascular dementia when cerebrovascular events were examined in a population-based study [71]. A more recent prospective study of 37,000 patients with a mean average age of 60 showed a significant increase in cognitive impairment incidence during a 5 year follow-up period [72]. 

Atrial fibrillation also appears to involve a significant conversion to dementia in nondemented subjects whether or not cognitive impairment was present [73]. Many studies have shown that atrial fibrillation induces significant brain hypoperfusion [74] which can compromise the aging cerebrovasculature. Although the true mechanism that associates atrial fibrillation to cognitive impairment is unclear, a suspicion is that cerebral hypoperfusion may be triggered by the chronic arrhythmia present [75]. 

## 6. Aortic and Mitral Valve Prolapse

Few studies have examined the effects of myocardial valve damage and its possible effect on cognitive function. Autopsy findings have reported significant aortic and mitral valve damage in AD subjects when compared to a nondemented control group [76]. This association between valvular damage and AD is consistent with the presence of brain hypoperfusion at an early stage of AD pathology [76], or even prior to AD, an observation that supports previous findings [77]. A more recent study revealed that left atrial fatigue which is a contributor of atrial fibrillation can develop from mitral valve damage involving either mitral valve prolapse or valvular regurgitation [78] (Figure 1).

Mitral valve prolapse and abundant senile plaques characteristic of AD were found at autopsy in the brains of elderly subjects who died from critical coronary artery disease but were not demented [78]. This finding was in contrast to nonheart disease subjects who showed almost no AD lesions when compared to heart disease patients [78]. The link between senile plaque formation and cardiac valvular damage implies that structural cardiac damage may influence the formation of neurodegenerative lesions even when no dementia has yet been expressed.

It is tempting to speculate that left atrial dysfunction stemming from mitral valve damage can be a therapeutic target that is preventable either by surgical or pharmacologic approaches to lessen the risk of AD in such patients. More work should determine whether this speculation has any merit.

## 7. Hypertension

According to the American Heart Association, some 60 million Americans have high blood pressure although about 70% of these show only mild hypertension. The incidence of hypertension in other countries such as China appears to be even higher due to their high salt intake. High blood pressure is estimated to affect 25% of the adult population in developed countries such as the USA and Canada. 

It is well established that high blood pressure can increase the risk of stroke and heart disease and decrease life expectancy. Many studies including the Framingham, the Kungsholmen, and the Honolulu-Asia Aging studies have implicated impaired cognitive function to hypertension in geriatric patients [79–81]. It has also been known for some time that hypertension in the elderly is a potential risk factor to AD [82–86]. 

The mechanisms linking hypertension to Alzheimer's disease remain to be elucidated, but damage to brain endothelial cells and smooth muscle cells that control cerebral blood flow is suspected to be a culprit due to the pulsatile pressure changes on the cerebral microvasculature generated by chronic hypertension [87]. These hemodynamic and structural changes produced on the brain by the heart can induce chronic brain hypoperfusion leading to white matter lesions as seen on cerebral magnetic resonance imaging of AD patients and constitute a good marker for this dementia [88]. 

When lacunar infarcts or accumulation of lesions in the white matter secondary to chronic hypertension lead to subsequent cognitive deterioration, it has been proposed that disconnection of cortico-subcortical pathways may be involved [89]. Possible neurotoxicity from the increased production of amyloid-beta_40_ in hypertensive patients may compromise cerebral perfusion by promoting vasoconstriction and/or cerebral angiopathy [90]. 

What is still not clear is precisely how hypertension increases the incidence of AD, particularly in those not treated with antihypertensives [80]. Also unclear is whether antihypertensive therapy can significantly reduce or reverse the cognitive decline that can herald dementia. We have theorized that chronic brain hypoperfusion generated by increased vascular resistance from hypertension may be a key factor linking high blood pressure and AD [91, 92]. Brain hypoperfusion resulting from hypertension can result from vessel stiffness secondary to atherosclerosis, increased vascular resistance, and disturbed hemodynamic flow patterns [17, 92] and is plausibly the basis for the gradual cognitive decline seen during advanced aging [93].

Recent randomized controlled trials including SYST-EUR, PROGRESS, HOPE, MRC, SHEP, SCOPE, and HYVET-COG examined the impact of antihypertensive therapy with cognitive function as a secondary end point. Three studies found positive results using antihypertensive therapy in preventing cognitive decline and dementia while the other four (MRC, SHEP, SCOPE, and HYVET-COG) trials reported no significant differences between treated and untreated subjects [94]. 

Since absence of evidence is not necessarily evidence of absence, a cautious conclusion can be advanced that the positive results of SYST-EUR, PROGRESS, and HOPE open the possibility of discovering target-specific cardiovascular therapies to lower AD and VaD prevalence by correcting the structural and physiological deficits encountered by a stressed heart.

For starters, a recent large population-based study of persons 65 years and older in Cache, Utah, reported that the use of antihypertensive medication, including angiotensin-converting enzyme inhibitors, *β*-blockers, calcium channel blockers, and diuretics significantly lowered the risk of AD [95]. The greatest reduction in AD risk (70%) was seen in the group using potassium-sparing diuretics, but the immediate reason for this finding was not clear [95]. It is possible that increased potassium levels are associated with a reduced risk of dementia [96] while low potassium concentrations have been associated with oxidative stress [97], aggregation [98], and vasoconstriction [99], conditions which can result in chronic brain hypoperfusion and, according to us, contribute to AD [100]. 

High blood pressure is known to reduce cerebral blood flow but the mechanisms involved in this action remain unexplained [57]. Since cardiac output remains normal during high blood pressure, two possible causes for the cerebral blood flow reduction may occur in hypertensive individuals. First, hypertension can increase systemic vascular resistance and slow down normal blood flow. Second, cerebral blood flow may fall when blood pressure is high due to direct damage to brain endothelial cells that produce the vasodilator nitric oxide [101]. Thus, there is an implied possibility that therapy aimed at controlling high blood pressure using potassium-sparing diuretics, increasing cerebrovascular nitric oxide, or protecting endothelial cells in the brain could prevent or delay the onset of AD by counteracting the appearance of chronic brain hypoperfusion in the hypertensive population [92]. In fact, increasing vascular nitric oxide in brain should provide another benefit. Vascular nitric oxide activation has been shown to downregulate BACE-1, the initial proteolytic enzyme responsible for A*β* peptide synthesis, while upregulating BACE-2, the enzyme that cleaves amyloid precursor protein (APP), so that A*β* production cannot take place [102]. 

## 8. Hypotension

Hypotension is a common clinical condition that commonly affects elderly persons over age 70. The most common causes of hypotension are blood pressure anomalies, dehydration, bleeding, medications, genetics, and cardiac pathology, including carotid sinus and vasovagal syndromes. 

Low diastolic blood pressure is associated with an increased risk of AD in the elderly population, particularly among users of antihypertensive drugs. While the reason for this finding is not clear, undetected cerebral hypoperfusion could explain the pathogenic link of hypotension to cognitve decline and AD [35]. 

In the Baltimore Longitudinal Study of Aging, both hypertension and hypotension have been shown to be associated with poorer performance on tests of executive function in older individuals and perceptuomotor speed and confrontation naming among persons not receiving antihypertensives medication [10]. 

Although the collective evidence appears to support a strong relationship between hypertension and the development of AD or VaD [103], no definitive evidence has yet been presented that antihypertensive treatment is a preventive measure to cognitve decline or dementia in people with high blood pressure, and the mixed evidence obtained in the seven randomized clinical trials discussed above is a glowing example. This lack of definitive evidence must be weighed in terms of the effects of iatrogenic-induced hypotension when aggressive anti-hypertensive therapy is used. 

For example, a common office procedure that has received almost no clinical attention in relation to AD development may be cited. That procedure is “white coat hypertension” (WCH). WCH occurs when a transient and usually mild increase in blood pressure is observed in certain individuals when attending a doctors' office. WCH is not generally linked to target organ damage or prognosis from true normotensives [104]. 

Although there is a controversy about whether WCH should be treated or not, many doctors assume mild blood pressure elevation needs to be treated, especially in the elderly. As a consequence, these individuals are given antihypertensives for the rest of their lives which can lower their blood pressure subnormally. It must be remembered that in the aging individual, diastolic hypotension can not only introduce cerebral hypoperfusion, but also the prospect of AD [9]. The best way to avoid falsely treating WCH is for physicians to place the new patient on the recumbent position and measure blood pressure several times during a 30 minute visit. 

The other side of the coin is not treating moderate or high hypertension or preventing hypotension, and this choice can lead to cardiac and cerebrovascular complications and the threat of death. For these reasons, more studies are rapidly needed that can guide the practitioner in managing a patient with blood pressure anomalies.

## 9. Heart Failure

Heart failure is a condition in which the heart can not adequately pump enough blood to meet the body's needs. It is marked by weakness, tissue edema, and shortness of breath. The most common cause of heart failure occurs from stenosis of the coronary arteries which supply oxygen to the heart.

Heart failure is often associated with other comorbid vascular risk factors for AD including ischemic heart disease, hypertension, and atrial fibrillation [45]. Brain hypoperfusion is a common outcome of heart failure [9].

As blood flow pumped out of the heart slows down, returning venous blood to the heart backs up, causing tissue edema particularly in the lungs. Heart failure is the most common reason for hospitalization among older adults [105] and has been reported to worsen cognitive impairment [106] and increase the risk of AD.

A recent report by Alves and her colleagues [107] indicates that heart failure in elderly persons is associated with lowered cerebral blood flow in the posterior cingulate gyrus and the lateral temporoparietal cortex, regions that are linked to memory and visuospatial orientation. This finding is of interest since memory and visuospatial dysfunction are two of the earliest signs in imminent AD. Although CBF and cognitive function is reduced in heart failure, this syndrome can improve following implantation of a pacemaker in patients with bradycardia or from the use of selective cardiovascular agents [108]. Clearly, aggressive treatment of heart failure to reverse brain hypoperfusion could have a significant impact in reducing the incidence of AD in these patients.

## 10. Coronary Artery Disease (CAD)

Coronary artery disease (CAD) is the single leading cause of mortality in the United States, resulting in over 900,000 deaths annually. CAD is associated with a decreased blood supply to the heart, also known as ischemic heart disease. This happens when the arteries that supply blood to the heart muscle become hardened and narrowed due to a build-up of subintimal fatty deposits called plaques. These atheromatous plaques are made up of a chemical bouillabaisse that includes cholesterol, fatty compounds, inflammatory cells, calcium, and fibrin. Plaques are the basis of atherosclerosis in coronary, peripheral, or cerebral blood vessels. When a plaque suddenly ruptures, platelets aggregate around it inducing intraluminal thrombosis or increased narrowing of the vessel, a condition that can result in myocardial infarction or stroke. The use of platelet deaggregators such as aspirin and clopidogrel or cilostazol, a phosphodiesterase type 3 inhibitor that can widen vessel lumen to increase blood flow, has been used to prevent cognitive dysfunction after intraarterial plaque rupture. Stroke and CAD are known to reduce brain blood flow and potentially impair cognitive function, and both are reported to be vascular risk factor for AD [78, 109]. 

The risk to AD could stem primarily from atherosclerotic coronary vessels that damage endothelial cells and lower the heart's pumping ability to optimally perfuse the brain. This thinking is supported by the presence of high levels of cholesterol, low density lipoprotein and triglycerides found in the blood of probable AD subjects [110]. 

A number of studies are now in progress testing whether cholesterol homeostasis and lipoprotein disturbances using cholesterol-lowering statins can alter AD pathology. However, the results of these studies are inconclusive and controversial with regard to the potential neuroprotective effects of statins [111]. Another approach we and others have recommended is to pharmacologically increase endothelial vascular nitric oxide, a powerful vasodilator with antithrombotic, anti-ischemic, and antiatherosclerotic activities [112]. 

## 11. ApoE_**4**_ Allele

Carriers of the ApoE_4_ allele may be at higher risk of cognitive decline because among other things, the presence of this gene predisposes to an increased risk of cardiovascular pathology [113]. 

Aside from sharing many environmental risk factors for AD and for cardiovascular disease, there appears also to be an overlap between genetic risk factors for both conditions. For example, the ApoE_4_ allele, a well-studied risk factor for AD, can increase the risk of coronary heart disease by approximately 40% [114].

Although the functional activity of ApoE_4_ varies considerably, one association found in the Baltimore Longitudinal Study of Aging was that carriers of this genotype but not noncarriers had greater decline in cerebral blood flow in nondemented older adults [115]. This decline in cerebral blood flow was observed in the frontal, parietal, and temporal cortices, which are the common brain regions initially affected in Alzheimer's disease [115]. 

## 12. Aortic Stiffening

Aortic stiffening is associated with advanced aging and hypertension (116ribkin). There is also compelling data that links aortic stiffening in the elderly to cognitive dysfunction (117, 118/mehra/hanon). Evidence indicates that cognitive impairment induced by damaged cerebral microcirculation can be induced by aortic stiffness [116]. The damage to brain microvessels from aortic stiffening would occur as follows. The aorta is known to be a reservoir of pulsatile energy delivered by left ventricular ejection during systole and discharges that energy during diastole. 

Since the aorta is normally more compliant (distensible) than the stiffer carotid arteries, it is believed to absorb the ventricular ejection and dampen pulsatile flow into the distal vasculature, a hemodynamic condition called the Windkessel effect [117]. The Windkessel effect is a protective mechanism that dampens excessive transmission of pulsatile flow that can damage the cerebral microvasculature [118]. The normal proximal aorta consequently reduces aortic-carotid wave reflection, a physiologic event called impedance mismatch, that avoids excess transmission to the cerebral arterioles and capillaries.

However, during aging, hypertension, or atherosclerosis, there is a loss of elastin which provides elasticity to the aorta, markedly reducing aortic compliance with the net effect of increasing pulse pressure and systolic pressure (hypertension) and reducing wave reflection at the carotid arteries. This hemodynamic phenomenon is worsened in the presence of vascular risk factors [118] (Figure 2).

When this happens, exaggerated pulsatile flow or pulse wave velocity is transmitted to microvessels in the brain where white matter hyperintensities and damage to endothelial cells that participate in controlling cerebral blood flow may result [119]. This pathologic event assumes a mechanistic link between cerebral hypoperfusion and cognitive impairment whose primary trigger is loss of the Windkessel effect [118]. Progressive increases of pulse wave velocity are theorized to result in increasing cognitive decline and eventual AD [120]. 

## 13. Cardiovascular Pathology Begets Chronic Brain Hypoperfusion Which Begets Progressive Cognitive Decline

Chronic brain hypoperfusion is not a disease but a sign that cerebral perfusion is functioning improperly. Cerebral blood flow is known to decline with aging and under normal circumstances in the absence of vascular disease will not result in significant cognitive loss [62]. To appreciate the clinical importance of developing suboptimal brain blood flow during advanced aging, it is important to point out that chronic brain hypoperfusion has been reported within the last few years to be a preclinical condition to mild cognitive impairment and a most accurate indicator for predicting whether people will develop AD [5, 62–64, 73]. 

The vascular hypothesis of Alzheimer's disease (AD), which we proposed in 1993 [121], has become a mother lode of interdisciplinary research involving mainly the brain, the heart, and the circulation [122–128]. The collective evidence supporting the vascular hypothesis offers the possibility of employing interventions that limit the effects of vascular risk factors on the heart and brain. This action could prevent, delay, or reverse further progression of the inherent cognitive deterioration that often precedes AD and VaD [129].

Following our vascular hypothesis proposal in 1993 [121] we observed in1994 [130] that conditions such as advanced aging, a former head injury, and apoE_4_ genotype became *risk factors* to AD by virtue of their potential to lower blood flow to the brain. Since then, several dozen heterogenous vascular risk factors to AD have been reported in the literature [16, 64, 73, 131–135]. 

But, if chronic brain hypoperfusion is initially involved in AD, how does cardiovascular disease become a risk factor for AD?

We believe that cardiovascular disease and the risk factors that characterize it promote brain hypoperfusion in the aging individual by inducing cerebral hemodynamic deficits and reducing blood flow to the brain through various vasculopathic pathways [17] (Figure 2). One likely pathway is the further burden that vascular risk factors add to the already reduced cerebral blood flow that is present as a result of aging [22–26]. 

This double burden on blood flow can readily lead to a neuronal energy crisis characterized by a cerebral hypometabolic state that can usher cognitive decline and dementia [136]. 

The neuronal energy crisis is typically followed by progressive neuronoglial dysfunction and eventual neuronal death. The *crisis-dysfunction-death *spectre begins in ischemic-sensitive zones such as the hippocampus and specific cortical areas [127] and will be clinically expressed initially by mild memory impairment [137] (Figure 2).

A relentless and progressive brain hypoperfusion can then spread to other parts of the brain where more ischemic-resistant neurons are slowly destroyed. This action is seen to spin out of control when additional cognitive impairment becomes full-blown AD. 

## 14. How Neuronoglial Energy Crisis Leads to AD Pathology

An eloquent example of how dependent neurons and glia are metabolically coupled to regional brain blood flow is shown in a PET study using [^18^F]fluoro-2-deoxyglucose that mapped cerebral blood flow in *normal *human brain. That study found that within a vascular territory, measures of cerebral blood flow and glucose metabolic rate are practically linear [138]. Moreover, this study revealed that the cerebellum, despite its significantly lower metabolic activity relative to the hippocampus, is as richly perfused as the hippocampus [138]. This finding is strikingly compelling in explaining the subcellular changes and markers of damage that occur in the hippocampal neurons but not in the less-active cerebellar neurons prior to AD. What may occur here is that a neurono-glial crisis begins to build up following chronic brain hypoperfusion which in time is expressed by abundant deposition of amyloid-beta containing plaques (Abeta) and neurofibrillary tangles (NFTs) in the hippocampus (an area critical for learning and memory) but basically spares the cerebellum, even at an advanced stage of AD. This difference between abundant AD lesions in the hippocampus but not in the cerebellum is dependent on the separate metabolic activity discharged by each neuronal population and the fact that energy supply and demand is greater in the hippocampus than in the cerebellum. These diverging metabolic activities expressing neuronal death markers in the hippocampus but not in the cerebellum may be due to two pathologic events occurring prior to AD neurodegeneration. 

The first pathologic event explains how cardiovascular disease is capable of inducing brain hypoperfusion and become an AD risk factor during aging. Although energy consumption is much greater in the hippocampal neurons than those in the cerebellum, the glucose energy supply is similar to both neuronal populations. However, while the cerebellum is allowed to keep its neurons well-fed energetically, even when glucose delivery is reduced due to cardiovascular insufficiency, the hippocampal neurons struggle to survive with the same glucose deficiency that is parsimoniously available to all neurons from the hypoperfused state. This *selective *brain cell energy crisis is the direct result of *blood flow supply not meeting energy demand* in highly metabolically active neurons whose vascular reserve capability has reached its limits from persistent cerebral hypoperfusion [139]. At this stage, hippocampal and cortical neurons undergo oxidative and endoplasmic reticulum stress that provide less ATP, the main energy fuel for cells [139], necessary to maintain normal function required for cell survival. This activity negatively affects posttranslation processing steps resulting in impaired protein transport, synthesis, assembly, and folding. Defects occurring during their synthesis, assembly, or folding can compromise the normal intracellular and extracellular secretory transport pathway, threatening brain cell survival that results in progressive cognitive decline [46]. 

A fundamental principle in cell biology is seen by the use of chemical energy in the form of ATP to assemble, disassemble, and alter protein structure. Since proteins do most of the work to keep neurons healthy, their proper production and folding are crucial for normal behavior, particularly involving learning and memory. Protein misfolding is a process in which proteins are unable to attain or maintain their biologically active shape [140]. Most proteins require assistance from molecular chaperones for proper folding [141]. These chaperones are specialized proteins which protect other unfolded proteins from misfolding and clumping (aggregating) together extracellularly (Figure 2). Although protein folding is thought to be a spontaneous process not requiring energy input from nucleotide triphosphates [142], the steps leading to it, involving transcription, translation, and protein synthesis, are energy dependent [143, 144]. Given the complexity of the folding process, it is not surprising that things can go wrong particularly in the presence of reduced cerebral perfusion and lowered energy substrate delivery during advanced aging.

Defects occurring during protein synthesis, assembly, or folding can compromise the normal intracellular and extracellular secretory transport pathway, threatening brain cell survival that results in progressive cognitive decline [46]. 

Protein cleavage abnormalities and reduced degradation of oxidized proteins by the ubiquitin-proteasome pathway may facilitate BACE-1 expression, the proteolytic enzyme responsible for generating Abeta peptide [145]. This process could also downregulate BACE-2, the enzyme that cleaves *β*-amyloid precursor protein at a site that prevents Abeta production [145]. Other subcellular aberrations from reduced ATP synthesis include (among other things) neurotransmitter failure, free radical production, Na^+^-K^+^ ATPase pump dysfunction, lower trophic/growth factor uptake, and faulty motor-protein transport within microtubules. This time-bound subcellular corruption climaxes with synaptic loss and neuronal death [146]. 

The second pathologic event explains why there is a scarcity of amyloid plaques and NFTs in the cerebellum while substantial aggregation is seen in the hippocampus of AD brains. During chronic brain hypoperfusion, the cerebellum enjoys all the glucose it needs to supply its less energy-consuming neurons, while the energy-starved and highly active hippocampal neurons undergo progressive subcellular changes due to their greater demand for energy supply. This energy supply/demand inequity consequently leads to the start of a neurodegenerative process involving the formation of intracellular-extracellular Aß and axonal NFTs in the hippocampal region while mostly sparing the cerebellum. The neuronal bias that generates an unequal energy crisis in brain regions of high metabolic demand such as the hippocampus and lower metabolic activity such as the cerebellum is a testament to the argument that amyloid plaque aggregation is a product (not a cause) of selective neuronal energy failure [134, 147]. Supporting this conclusion are studies by us [129, 148, 149] showing that after chronic brain hypoperfusion in aging rats, memory loss, and a selective reduction of cytochrome oxidase, reflecting lower ATP activity, was found uniquely in the CA1 region of the hippocampus and in the posterior parietal cortex, two regions associated with memory function and the initial targets of AD neurodegeneration [150, 151]. 

A more detailed description of this neuronal energy crisis inequity can be found in our previous publications [19, 100, 129, 146, 152]. 

Finally, much confusion has been generated surrounding the terms *cerebral hypoperfusion *and *cerebral ischemia*, when they have been used interchangeably in the literature. To avoid this confusion, the term cerebral hypoperfusion should be used to describe the relatively slow pathologic process involving months or years when brain perfusion is not commensurate with neurometabolic demand. Conversely, cerebral ischemia should refer to the more rapid pathologic process involving hours or days of sudden blood flow reduction that is capable of killing or damaging brain cells in the epicenter of the ischemic lesion and often allows the formation of a penumbral region made up of undamaged but inactive or “idling” neurons [121]. This definition of hypoperfusion and ischemia is useful in trying to explain the “slow” from the “fast” cognitive decline seen after either event, resulting for example, from the presence of long-term vascular risk factors (hypoperfusion) or a sudden stroke (ischemia). 

## 15. Slowing AD Prevalence

Can anything be done to slow down the total number of new AD cases expected in the next few decades? In our judgment [152–154] and those of others [153–155, 155–159] and in the absence of finding a rapid cure for this dementia, preventive measures to lower the prevalence rate of AD (and by default, VaD) through the management of potential or actual risk factors is a reasonable clinical strategy. A structured clinical approach can be employed for this purpose. It requires the diagnostic detection of cardiovascular disease and assessment of warning signs for stroke in elderly individuals (>60 years) who show or complain memory difficulties during clinical examination. It should be noted that ischemic heart disease or ischemic stroke are not the sole vasopathogenic triggers of AD. Many other vascular-related risk factors that increase the burden of age-related cerebral hypoperfusion also appear to accelerate the development of Alzheimer dementia, and these have been reviewed in other publications [6–8]. This is a theme that has not received wide-attention in the medical literature despite its obvious importance.

Based on cross-sectional and longitudinal epidemiologic studies involving mostly elderly subjects, a variety of cardiovascular-related risk factors to AD have been reported. These include high serum lipid/cholesterol levels, high serum homocysteine, body-mass index, smoking, physical inactivity, unhealthy diet, hypertension, hypotension, and metabolic syndrome [160]. Comorbid presence of two or more such risk factors tends to increase the probability of acquiring AD [161]. 

When any or several of these risk factors, are discovered during clinical examination, the physician should be suspicious of actual or impending structural heart damage. Structural heart damage may involve aortic or mitral valve thickening, chronic valvular regurgitation, left ventricular wall motion abnormalities, ventricular filling defects, and left ventricular hypertrophy. Structural heart damage can reduce cardiac output and ejection fraction (or cardiac index) thus directly affecting cerebral perfusion [162, 163]. In addition, cardiac damage resulting in hemodynamic pump dysfunction can be the source of ischemic stroke or cerebral hypoperfusion [47, 164] which in the elder population frequently evolves into cognitive impairment [165] and possible conversion to AD or VaD.

 Many of these cardiac risk factors, however, can be corrected or treated successfully if identified in time [166]. 

For example, high blood pressure is a major risk factor for stroke and for heart failure and as we have noted, is also closely correlated with cognitive decline and dementia. Its treatment with anti-hypertensive drugs in the elderly has been shown to reduce cognitive decline and dementia [167] and to partially counteract the risk of heart failure on dementia [168]. 

The heart-brain connection to memory function can be appreciated from experimental data on rodents. We reported that during exposure to chronic brain hypoperfusion in aged rats, vulnerable brain cells initially undergo a hypometabolic state that reduces memory function while neurons remain structurally intact [152]. Even after memory impairment is induced in these aged rats, the metabolically-compromised neurons can return to a normal state if and when cerebral perfusion is restored after 5 weeks following brain hypoperfusion [169]. These findings indicate two important points: (1) that cerebral hypoperfusion in these rodents can induce a hypometabolism that impairs memory function without killing the neurons, and (2) that neuronal rescue of the hypoperfused brain cells can be achieved even after a lengthy (by rat standards) period of reduced brain blood flow. If this rat model is indicative of that which may occur in elderly humans who develop chronic brain hypoperfusion prior to AD, a therapeutic target focusing on brain blood flow insufficiency could be a major breakthrough. The notion of neuronal rescue from the hypoperfused state is supported by clinical findings in human brain [170, 171]. It is also pertinent to note that brain hypoperfusion induced by cardiac pathology can promote not only VaD but also AD since these pathologic states pose important risk factors to both dementias [172]. 

## 16. Conclusions

We have attempted to show in the present paper the association between chronic brain hypoperfusion and cardiovascular risk factors of AD in a crystallized fashion pointing out the major problems associated with cardiovascular pathology, cerebral hypoperfusion, and development of AD. Cerebral hypoperfusion following cardiovascular pathology during aging is a much neglected research topic in AD that deserves greater attention. 

It is crucial to note that cardiovascular risk is generally present prior to AD and that AD neurodegenerative lesions such as Abeta and NFTs are not essentially considered precursors or triggers of heart disease but may be more a manifestation of the neuronal energy crisis provoked by vascular risk factors to dementia. 

The findings lend support to the design of therapeutic targets aimed at preventing chronic brain hypoperfusion and its consequential neural hypometabolism prior to the onset of cognitive symptoms or neuropathology during normal aging. The therapeutic blueprint will largely depend on early detection of low cerebral blood flow in asymptomatic individuals who present cardiovascular risks factors associated with progressive cognitve decline.
